# Spontaneous excretion of a pseudomembranous intestinal cast in an infant with an acute diarrhoeal illness: A case report and literature review

**DOI:** 10.1002/jpr3.12115

**Published:** 2024-07-31

**Authors:** David G. Cairney, Paul Fineron, Andrew J. Kirby, Paul Henderson, Peter M. Gillett

**Affiliations:** ^1^ Department of Paediatric Gastroenterology Hepatology and Nutrition, Royal Hospital for Children and Young People Edinburgh UK; ^2^ Pathology Department Western General Hospital Edinburgh UK; ^3^ Imaging Department Royal Hospital for Children and Young People Edinburgh UK; ^4^ Child Life and Health University of Edinburgh Edinburgh UK

**Keywords:** colitis, infantile diarrhoea, pseudomembrane

## Abstract

We present a case of an 8‐week‐old infant with acute bloody diarrhoea and subsequent passage of an intestinal cast. An extensive immune and infection work‐up did not reveal a causative aetiology. Histopathology indicated the cast represented an intestinal pseudomembrane. 16S bacterial polymersae chain reaction of the pathology specimen was negative. The infant required a period of parenteral nutrition due to diarrhoeal losses but made a full recovery and had no sequelae from this illness. Intestinal casts are a rare occurrence, particularly in paediatrics. It prompts a wide differential which includes acute infection, immunodeficiency and ischaemia. Accurate quantification of stool losses, appropriate nutrition support and liaison with microbiology colleagues were essential in this case.

## 1 INTRODUCTION

The passage of intestinal casts in all ages is a rare consequence of significant bowel injury with causes ranging from acute ischaemia to severe infection particularly in the context of immunodeficiency.^1^ We present a case of an intestinal cast which was histologically a pseudomembrane in a febrile infant with acute bloody diarrhoea.

## 2 CASE

**Figure 1:**
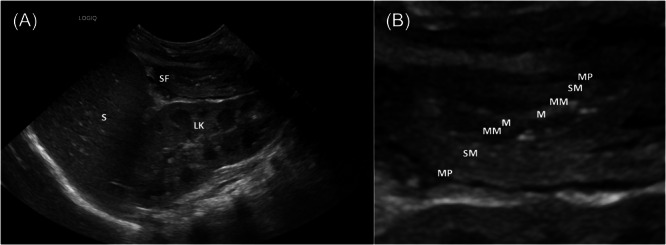
(A) Ultrasound image of the left upper quadrant on presentation imaging (Day 2) showing S, LK and SF. (B) Zoomed section from the same image showing the thickened splenic flexure of colon, indicating inflammation. The gut wall signature is outlined from innermost to outermost: echogenic M, hypoechoic MM, intermediate SM, hypoechoic MP. The serosa is not visible. The most inflamed layer at this time is the submucosa. The displayed left hemicolon was thick‐walled measuring up to 4 mm compared to the right hemicolon at 1.5 mm (not displayed). LK, left kidney; M, mucosa; MM, muscularis mucosa; MP, muscularis propria; S, spleen; SF, splenic flexure of colon; SM, submucosa.

An 8‐week‐old girl presented to hospital with a 24‐h history of watery stools and fresh red rectal bleeding. She was febrile at 38°C and irritable. Birthweight was on the 75th centile and had dropped to the 50th centile on a mixture of predominantly breast feeding with the recent introduction of formula milk top‐ups. There were no perinatal risk factors for infection or ischaemia. Her parents were non‐consanguineous with no relevant family history. She had not yet received her routine infant vaccinations. She had normal perfusion and a soft abdomen on examination. She underwent an infection screen including lumbar puncture, blood culture and virology testing (stool and respiratory polymersae chain reaction [PCR]). A broad spectrum intravenous (IV) cephalosporin was commenced. She initially continued milk feeds but was put nil by mouth on day three of admission due to persisting, profuse, diarrhoea and vomiting and IV fluids commenced. On Day 4, her stool output appeared to be improving so an amino acid feed was briefly trialled with recurrence of stool frequency and vomiting so this was stopped. On Day 5 she had an acute deterioration with pallor and low tone requiring fluid resuscitation. The lactate was normal and there was no concern regarding ischaemia. Bloods at this time revealed a haemoglobin of 107 g/L (normal range 111–141), an elevated white cell count of 30.2 × 10^9^/L (normal range 6.0–17.5) with a neutrophil predominance, a mildly elevated C‐reactive protein of 29 mg/L (normal range <5), hypoalbuminaemia at 23 g/L (no local reference range for this age group) and normal electrolytes. IV esomeprazole and metronidazole were added. An abdominal ultrasound revealed a thick‐walled colon and normal small bowel (Figure 1). Inotropic support was not required.

**Figure 2:**
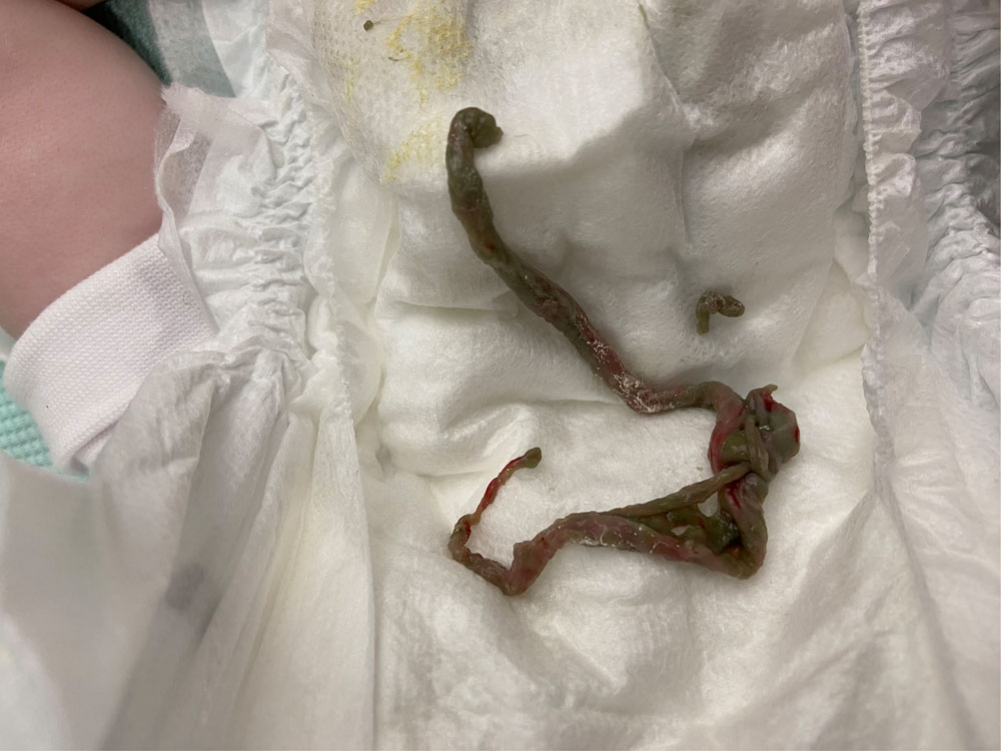
Spontaneously excreted cast‐like structure found in nappy on Day 6 of illness.

On Day 6 a spontaneously excreted intestinal cast was found in her nappy (Figure 2). There was no change in the stool output before and after excretion of the cast. Despite being nil by mouth from Day 4, her stool losses remained persistently high at up to 60 mL/kg/day so was commenced on parenteral nutrition via a peripherally inserted central line. She was catheterised to accurately assess urine (vs. watery stool) output and optimise fluid balance. Due to the high stool output, she remained nil by mouth until Day 11, at which point the stools abruptly decreased and she was weaned onto an extensively hydrolysed high‐energy feed over 8 days with no recurrence of diarrhoea. She was then successfully transitioned onto a whole protein formula and discharged home with normalising growth parameters. A timeline of her admission is available (Figure S1). At last follow‐up, she remained well with no concerns about growth, development or recurrent infection at 9 months. Repeat bloods showed normal vaccine responses (Haemophilus influenzae type b, Pneumococcus and Tetanus), immunoglobulins and routine biochemistry.

**Figure 3:**
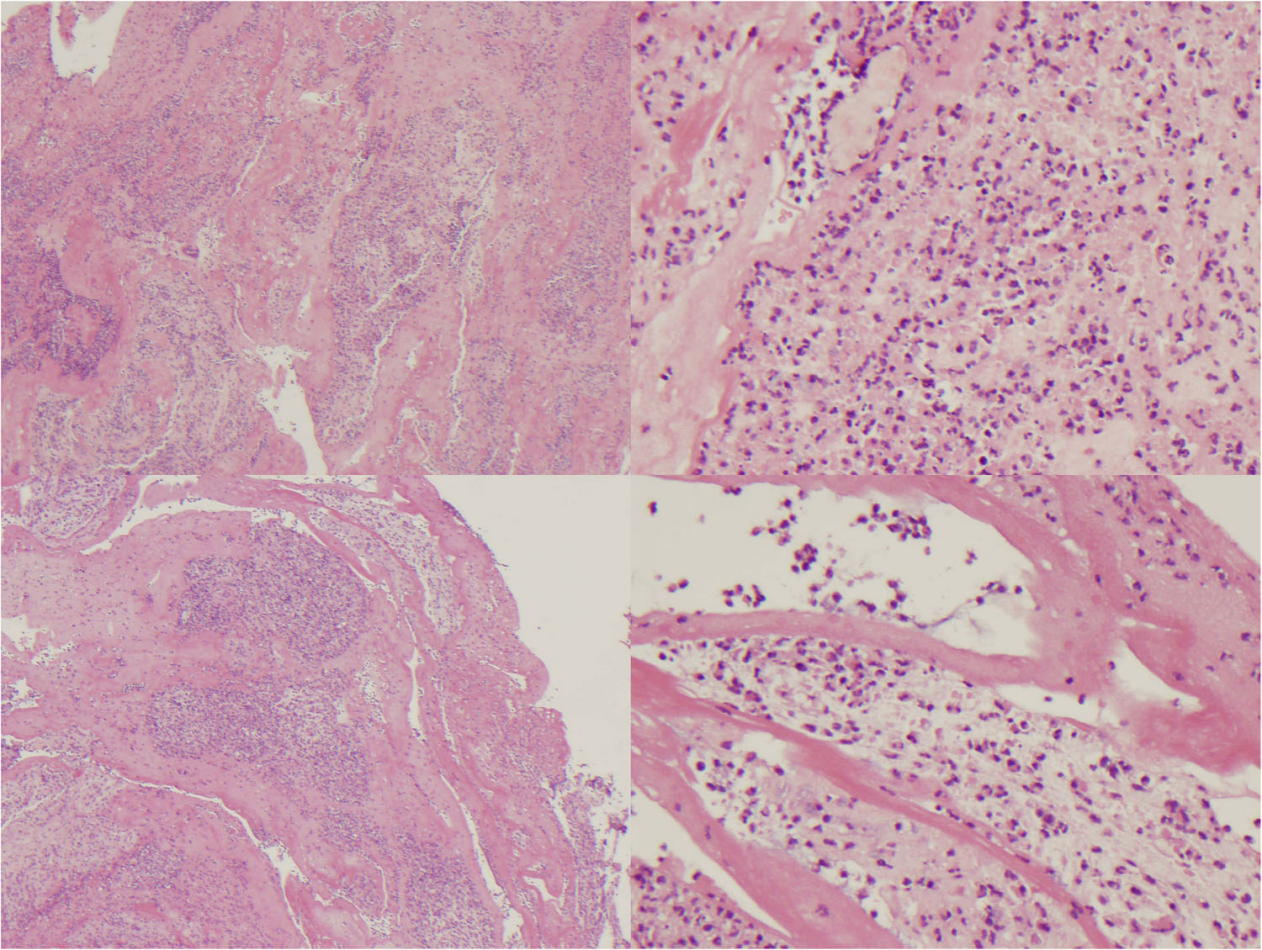
Acute fibrinopurulent exudate with small amounts of mucin and faecal debris. No evidence of colonic mucosa or deeper components of the colonic wall and no microorganisms. The appearances of this material are nonspecific. Admixture of fibrin and numerous neutrophils could represent a pseudomembrane.

Stool (adenovirus, parechovirus, rotavirus, sapovirus, norovirus and astrovirus), vomit (norovirus, rotavirus), respiratory (20 common respiratory viruses), blood (adenovirus, human immunodeficiency virus) and urine (cytomegalovirus) viral PCR were negative. Standard stool culture was negative (including Cryptosporidium/Giardia microscopy and Salmonella, Shigella, *Escherichia coli* 0157 and Campylobacter culture) but *Clostridium difficile* testing was only possible on stool samples obtained after antibiotics and this was negative. Blood, urine and CSF culture were also negative. An extensive immune work‐up (neutrophil respiratory burst, immunoglobins and lymphocyte subsets) only revealed a mildly elevated IgA. Histopathology of the cast (Figure 3) revealed an acute fibrinopurulent exudate in keeping with a pseudomembrane. A primary immunodeficiency genetic panel was normal (Table S1). Bacterial 16S rRNA PCR on the cast tissue was subsequently negative.

The need for endoscopic assessment was considered but she was initially too unstable to undergo anaesthesia and endoscopy and until the pathology was reported, it was considered the risk of endoscopy (and iatrogenic harm) outweighed the diagnostic benefits. Subsequently due to her clinical improvement it was deemed unnecessary.

## 3 DISCUSSION

We present an unusual case of an intestinal cast in an infant with acute diarrhoea. No causative pathogen or underlying risk factor were identified despite an extensive work up for infection and immunodeficiency. The mode of presentation and eventual resolution of symptoms after a period of gut rest on parenteral nutrition and antibiotics would be in keeping with an unidentified infective agent.

At the point of excretion of the cast, the assumption was that this represented denuded intestinal mucosa particularly given the high diarrhoeal losses. Intestinal casts are rarely reported in adult or paediatric literature and are most described post bowel surgery or in the context of intestinal ischaemia.^1^, ^2^

The paediatric literature is confined to three cases. The first was a 4‐year‐old being treated with amoxicillin for a chest infection who subsequently developed systemic upset, profuse diarrhoea and subsequently passed an intestinal cast. The histology was indicative of a pseudomembrane.^3^ *C. difficile* culture was negative. The second was a 6‐year‐old with fever, pain and refractory diarrhoea with previous episodes of similar symptoms. This was felt to represent an autoinflammatory disorder mimicking monogenic inflammatory bowel disease.^4^ The third was a 4‐year‐old girl with leukaemia who had received a cord blood transplant with subsequent gastrointestinal graft versus host disease despite immunosuppression, steroids and antibiotics.^5^ After passing a colonic cast, she was treated with hyperbaric oxygen therapy for 1 week.

Histopathology varies in reported cases from full‐thickness denuded intestinal mucosa to fibrinopurulent inflammatory material.^2^ The histopathology, in this case, raised the suspicion of pseudomembranous colitis as the aetiology. The clinical presentation raised a broad differential diagnosis which included acute infection, immunodeficiency and ischaemia. A standard infection screen was negative. *C. difficile* pseudomembranous colitis was raised as a potential diagnosis after the urgent pathology results were received. *C. difficile* has been regarded as a harmless gut commensal in this age group. It has been proposed that infants lack the toxin receptors necessary for developing severe infection, however, cases of symptomatic infection have been reported.^6^ It is not standard practice to test for *C. difficile* on infant samples at our institution, so this result was not available from preantibiotic samples. Bacterial molecular testing on the tissue sample was subsequently negative. Our case is truly idiopathic. A suggested workup is proposed for such cases (Table S2) which would include *C. difficile* and this may require specific liaison with microbiology specialists depending on local procedures. Careful attention to fluid balance and early catheterisation is key in such cases of infant diarrhoea in addition to the use of parenteral nutrition in the acute phase of the illness and recovery.

## CONFLICT OF INTEREST STATEMENT

The authors declare no conflict of interest.

## Supporting information

Supplementary figure 1: A timeline of the patient's admission. NBM ‐ nil by mouth, PN ‐ parenteral nutrition.

Supporting information.

Supporting information.
