# Supplementary material for: Spontaneous excretion of a pseudomembranous intestinal cast in an infant with an acute diarrhoeal illness: A case report and literature review
Source: JPGN Rep. 2024 Jul 31;5(4):497–500. doi: 10.1002/jpr3.12115 (PMC11600368; doi:10.1002/jpr3.12115)
Supplement: Supplementary file 2 — Supporting information. [file JPR3-5-497-s002.docx]

| **Supplementary table 1:** Primary immunodeficiency genetic panel undertaken | |
| --- | --- |
| Next generation sequencing of coding regions and immediate flanking regions for the following genes: | RFXANK, CIITA, RFX5, RFXAPMAGT1, LCK, UNC119CD8A, ZAP70, TAP1, TAP2, TAPBP, B2MDOCK8, STK4, IL21, MPA3K14, MSNCD3G, RHOH, TRAC, BCL11B, OX40 (TNFRSF4), LATDOCK2, CARD11, BCL10, IKBKB, ICOS, TFRC, RELB, CD40, CD40LGIL21R, MALT1, IL10, IL10RA, IL10RB, NFAT5, ITGB2 (LAD I), SLC35C1 (LAD II), FERMT3 (LAD III), CYBB, NCF1, CYBA, NCF4 NCF2 |
