# Supplementary material for: Spontaneous excretion of a pseudomembranous intestinal cast in an infant with an acute diarrhoeal illness: A case report and literature review
Source: JPGN Rep. 2024 Jul 31;5(4):497–500. doi: 10.1002/jpr3.12115 (PMC11600368; doi:10.1002/jpr3.12115)
Supplement: Supplementary file 3 — Supporting information. [file JPR3-5-497-s001.docx]

| **Supplementary table 2:** Investigations in paediatric intestinal casts | |
| --- | --- |
| **Infection** | Stool culture including C diff toxin and parasite microscopy  Stool virology  Urine, blood or intestinal biopsy CMV PCR  HIV test |
| **Immunology** | Immunoglobulins  Lymphocyte subsets  Neutrophil respiratory burst  Primary immunodeficiency genetic panel |
| **Ischaemia** | Blood lactate  Abdominal ultrasound scan  Contrast enhanced CT |
